# Supplementary figures and images for: FSTL1 promotes dendritic cell pyroptosis and immunosuppression in sepsis by inhibiting STING autophagy
Source: PLoS One. 2026 Feb 17;21(2):e0340204. doi: 10.1371/journal.pone.0340204 (PMC12912587; doi:10.1371/journal.pone.0340204)

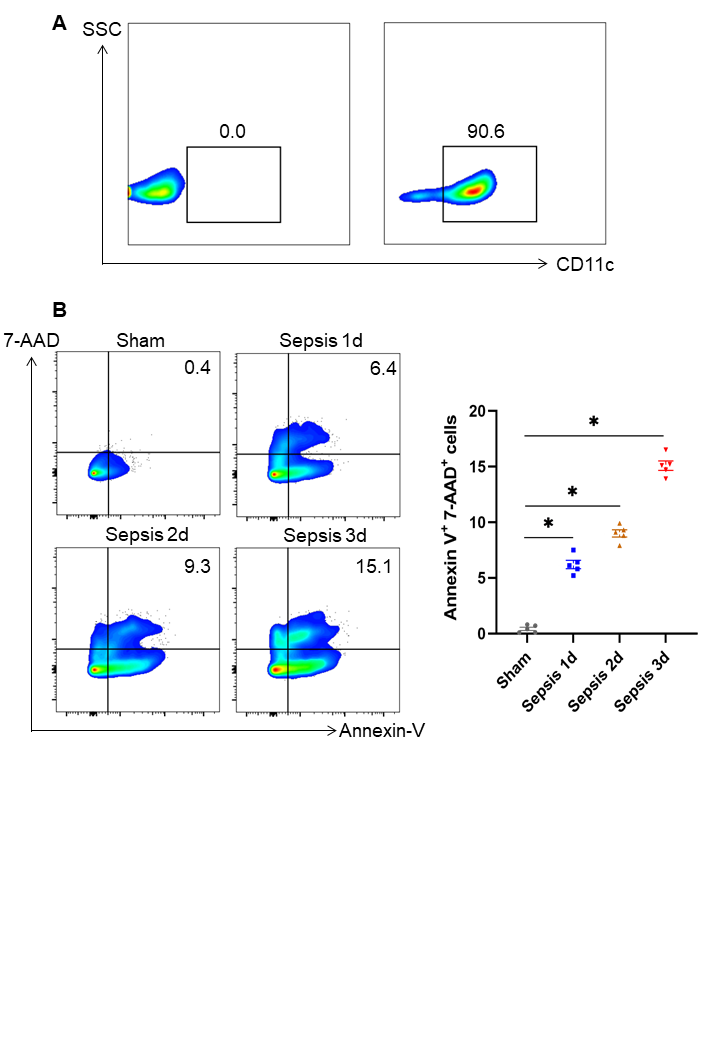

Supplement: S1 Fig — (a) The purity analysis of the splenic CD11+ DCs isolated from mice. (b) The pyroptosis rate of DC was calculated using flow cytometry. Data were represented as Mean ± SD, *P < 0.05. (TIF) [file pone.0340204.s001.tif]

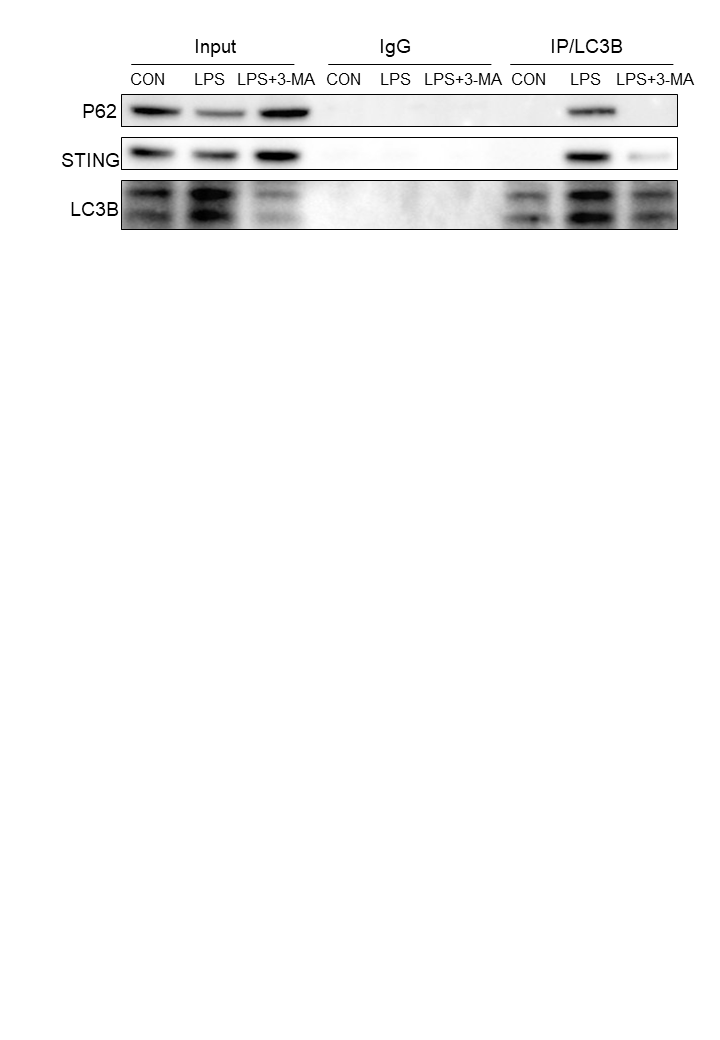

Supplement: S2 Fig — DC2.4 cultured in vitro were exposed to the corresponding solvent (control), LPS, or LPS + 3-MA for 24h. LC3B was immunoprecipitated from whole cell lysates of DC2.4, and levels of STING, LC3B, and P62 in the precipitates were evaluated by immunoblotting. (TIF) [file pone.0340204.s002.tif]

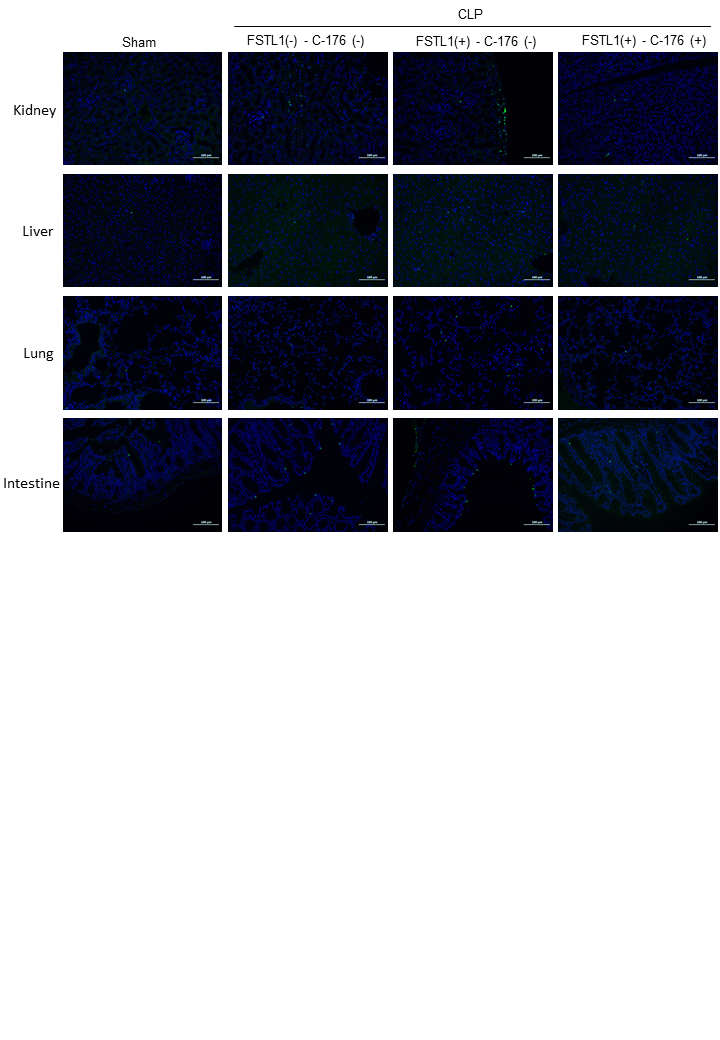

Supplement: S3 Fig — C57BL/6 mice were subject to sham or CLP operations and were given corresponding solvent (control), FSTL1 (10 mg/kg), FSTL1 + C-176 (20 mg/kg) by intraperitoneal injection. Kidney, liver, lung and intestine were collected 24 hours post-treatment and subjected to TUNEL staining. (TIF) [file pone.0340204.s003.tif]

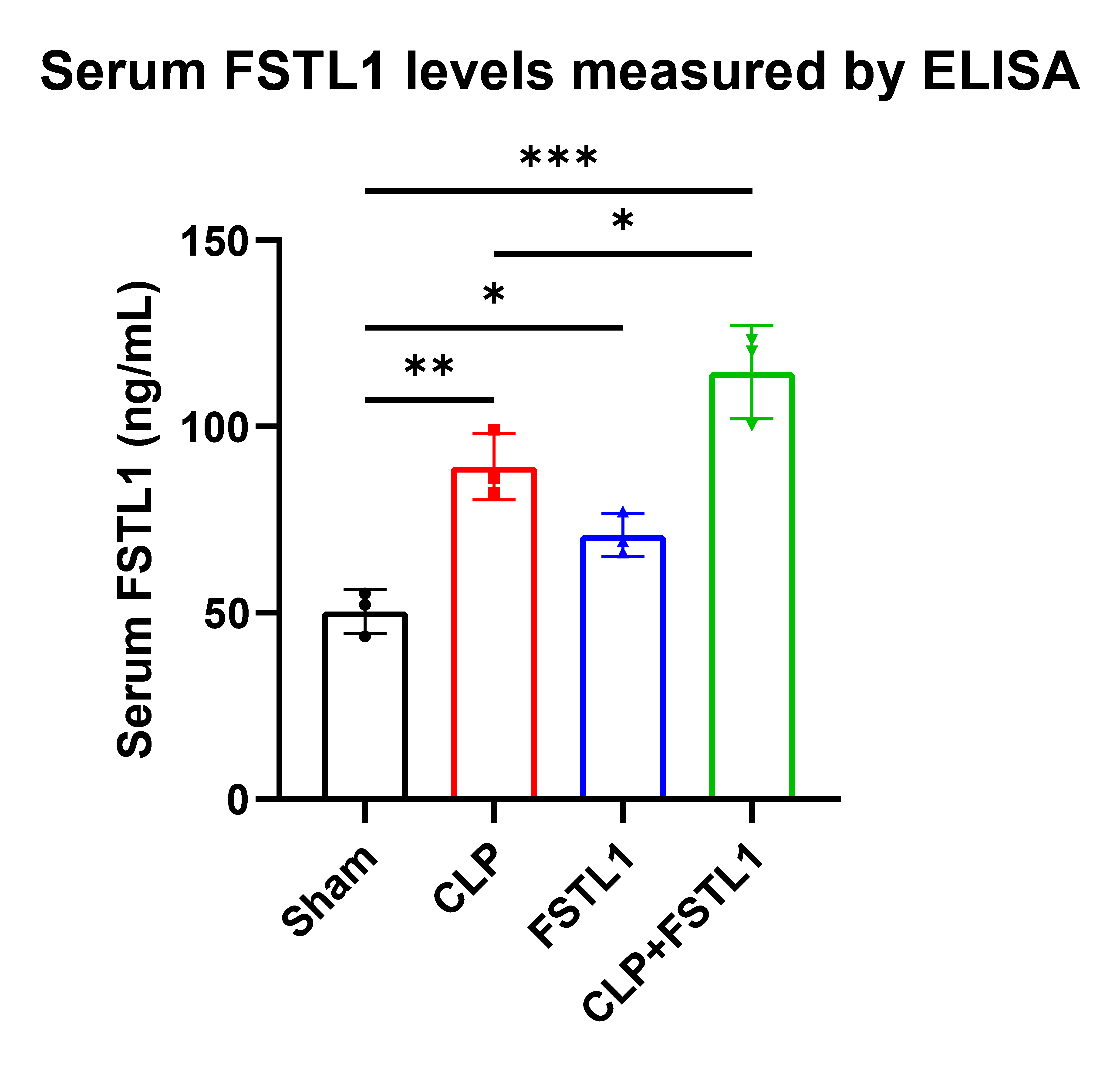

Supplement: S5 Fig — C57BL/6 mice were divided into four groups: Sham, CLP, FSTL1 (10 mg/kg), and CLP + FSTL1 (10 mg/kg) (n = 3). Serum FSTL1 concentrations were measured by ELISA 24 hours after treatment. FSTL1 levels were increased in septic mice compared with sham controls, moderately elevated in the FSTL1 group, and further increased in the CLP + FSTL1 group. (TIF) [file pone.0340204.s005.tif]
