# Supplementary material for: FSTL1 promotes dendritic cell pyroptosis and immunosuppression in sepsis by inhibiting STING autophagy
Source: PLoS One. 2026 Feb 17;21(2):e0340204. doi: 10.1371/journal.pone.0340204 (PMC12912587; doi:10.1371/journal.pone.0340204)
Supplement: S1 Table — (DOCX) [file pone.0340204.s006.docx]

Supporting information

S1 Table. List of antibodies.

| **Items** | **Company** | **Cat #** | **Assay (Dilution)** |
| --- | --- | --- | --- |
| Caspase-1 | Abcam | ab179515 | 1:1,000 (WB)  1:200 (IF) |
| GSDMD | Abcam | ab209845 | 1:1,000 (WB) |
| GSDME | Abcam | ab215191 | 1:1,200 (IF) |
| LMAN1 | Abcam | ab125006 | 1:200 (IF) |
| Caspase-3 | CST | #9662 | 1:200 (IF) |
| STING | CST | #13647 | 1:1,000 (WB)  1:200 (IF) |
| p-STING | CST | #19781 | 1:1,000 (WB)  1:200 (IF) |
| β-actin | Sigma-Aldrich | A5441 | 1:1,000 (WB) |
| IL-1β | ELK | ELK1271 | 1:1,000 (WB) |
| pTBK1 | Immunoway | YM8322 | 1:1,000 (WB) |
| TBK1 | Immunoway | YM8674 | 1:1,000 (WB) |
| LC3B | Immunoway | YM8147 | 1:1,000 (WB)  1:200 (IF)  1:100 (IP) |
| P62 | Immunoway | YM8025 | 1:1,000 (WB) |
| FSTL1 | Abcam | Ab22328 | 1:200 (IF) |
| IRF3 | Immunoway | YM8227 | 1:200 (IF) |
